# Novel approaches to minimize ventilator-induced lung injury

**DOI:** 10.1186/1741-7015-11-85

**Published:** 2013-03-28

**Authors:** Eddy Fan, Jesus Villar, Arthur S Slutsky

**Affiliations:** 1 Interdepartmental Division of Critical Care Medicine, University of Toronto, Toronto, Canada; 2 Department of Medicine, Mount Sinai Hospital and University Health Network, Toronto, Canada; 3 CIBER de Enfermedades Respiratorias, Instituto de Salud Carlos III, Madrid, Spain; 4 Research Unit, Hospital Universitario Dr Negrin, Las Palmas de Gran Canaria, Spain; 5 Keenan Research Center at the Li Ka Shing Knowledge Institute, St. Michael’s Hospital, Toronto, Ontario, Canada

**Keywords:** Acute lung injury, Acute respiratory distress syndrome, Critical illness, Cytokines, Extracorporeal membrane oxygenation, Heat shock response, Mechanical ventilation, Ventilatory support

## Abstract

Despite over 40 years of research, there is no specific lung-directed therapy for the acute respiratory distress syndrome (ARDS). Although much has evolved in our understanding of its pathogenesis and factors affecting patient outcome, supportive care with mechanical ventilation remains the cornerstone of treatment. Perhaps the most important advance in ARDS research has been the recognition that mechanical ventilation, although necessary to preserve life, can itself aggravate or cause lung damage through a variety of mechanisms collectively referred to as ventilator-induced lung injury (VILI). This improved understanding of ARDS and VILI has been important in designing lung-protective ventilatory strategies aimed at attenuating VILI and improving outcomes. Considerable effort has been made to enhance our mechanistic understanding of VILI and to develop new ventilatory strategies and therapeutic interventions to prevent and ameliorate VILI with the goal of improving outcomes in patients with ARDS. In this review, we will review the pathophysiology of VILI, discuss a number of novel physiological approaches for minimizing VILI, therapies to counteract biotrauma, and highlight a number of experimental studies to support these concepts.

## Background

Acute respiratory distress syndrome (ARDS) is defined by the acute onset of hypoxemic respiratory failure with bilateral infiltrates on chest radiography due primarily to non-cardiogenic pulmonary edema [1]. Despite intense research over four decades, no effective pharmacological therapies exist for ARDS, and supportive care with mechanical ventilation (MV) remains the cornerstone of treatment [2]. Perhaps the most important advance in ARDS research has been the recognition that MV, although necessary to preserve life, can itself aggravate or cause lung damage through a variety of mechanisms collectively referred to as ventilator-induced lung injury (VILI) [3]. These mechanisms include exposure to high inflation transpulmonary pressures (barotrauma), alveolar overdistention (volutrauma), and/or repetitive opening and closing of alveoli (atelectrauma). In addition to direct structural damage, these mechanical forces can trigger a complex array of inflammatory mediators, resulting in a local and systemic inflammatory response (biotrauma) propagating injury to non-pulmonary organs [4], which may result in multiple system organ dysfunction, and ultimately in death.

**Figure 1:**
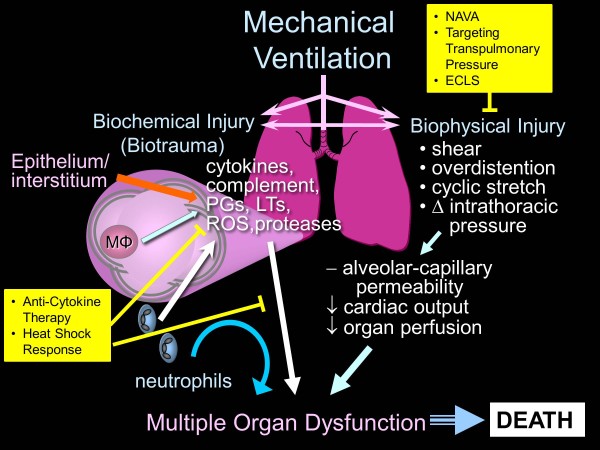
**Mechanisms for VILI and potential targets for physiologic and non-physiologic interventions to minimize VILI. **Reprinted with permission of the American Thoracic Society. Copyright (C) 2013 American Thoracic Society. Slutsky AS, Tremblay LN: **Multiple system organ failure: is mechanical ventilation a contributing factor? ***Am J Resp Crit Care Med *1998, **157:**1721**–**1725. ECLS, extracorporeal life support; LT, leukotrienes; MΦ, macrophages; NAVA, neurally adjusted ventilatory assist; PG, prostaglandins; ROS, reactive oxygen species; VILI, ventilator-induced lung injury.

This improved understanding of ARDS and VILI has been important in designing lung-protective MV strategies to attenuate VILI and improve survival. Indeed, the only strategy that has demonstrated improved survival in patients with ARDS is the use of low tidal volume (VT) (≤6 ml/kg predicted body weight (PBW) ventilation, along with adequate positive end-expiratory pressure (PEEP), and limiting plateau pressure to ≤30 cm H_2_O [5]. Although this strategy aims to minimize VILI due to volutrauma or atelectrauma, recent studies have revealed that tidal hyperinflation may occur despite the use of this strategy and there may be advantages to reductions below 6 ml/kg PBW [6], even if plateau pressures are <30 cm H_2_O [7]. Considerable effort has been made to enhance our mechanistic understanding of VILI and to develop new ventilatory strategies and therapeutic interventions to prevent and ameliorate VILI, and to improve outcomes in patients with ARDS. In this, we will briefly review the pathophysiology of VILI, and discuss a number of novel physiological and non-physiological approaches for minimizing VILI (Figure 1).

### Pathophysiology of VILI

VILI is not a new concept. The term historically applied to macroscopic injuries associated with alveolar rupture due to overdistension when high inspiratory pressures are applied. The term VILI has shifted somewhat from referring to pressure-induced (really, volume-induced) injury to increased permeability, accumulation of lung fluid, atelectrauma, and inflammation induced by MV [3]. VILI can resemble ARDS and it is difficult to diagnose in humans because its appearance can be similar to the underlying disease for which MV was instituted.

The main mechanical determinant of VILI is regional lung overdistention due to high transpulmonary pressure (stress) that causes the lung to deform above its resting volume (strain) [8]. In experimental models, VILI develops when a lung strain (estimated as the ratio between lung volume change and resting volume) greater than 2 is achieved, corresponding to a VT approximately greater than 20 ml/kg in healthy animals [8,9]. Thus, the smaller the resting lung volume, the greater the strain for a given lung volume change (inflation). MV at low lung volumes may also be deleterious, due to regional amplification of forces and repetitive opening and closing of distal, collapsed lung units [10,11].

VILI is a dynamic process that is hard to capture at a single time point. The damage observed in VILI reflects the primary injurious stimuli and the secondary complex interactions of inflammatory mediators on alveolar epithelial and capillary endothelial cells. Alveolar overinflation elicits a well coordinated response that contributes to cellular proliferation and inflammation. Lung cell deformation by mechanical forces originating within the alveolus direct conformational changes in molecules within the cell membrane, leading to activation of downstream messenger systems. MV can trigger a complex array of proinflammatory and anti-inflammatory mediators that may lead to either greater injury or enhanced lung healing and quicker restoration of pulmonary function [12]. Two mechanisms are believed to be responsible for this MV-induced inflammatory response (biotrauma). The first is direct trauma to the cell with disruption of cell walls, resulting in the release of cytokines into both the alveolar space and the systemic circulation [13]. The second mechanism has been termed mechanotransduction. *In vitro* studies have shown that most pulmonary cells can produce cytokines in response to cyclic stretch [14]. A large number of genes differentially expressed in the lung by MV have been identified in *in vivo* animal models of VILI, including genes involved in immunity and inflammation, the stress response, metabolism, and transcription processes [15]. However, the sensing mechanism of these physical forces and the translation into intracellular signals is largely unknown. Slutsky and Tremblay [16] were the first to suggest that the MV-induced inflammatory response may contribute to development of multiple system organ dysfunction seen in mechanically ventilated patients with ARDS by initiating or propagating a malignant, systemic inflammatory response. Although it remains unclear how inflammatory mediators exert their detrimental effects on distal organs, experimental studies and clinical trials in ARDS have shown that the application of protective ventilator strategies are associated with decreased serum cytokine levels [17,18], decreased extrapulmonary organ dysfunction [19], and decreased mortality [5].

### Physiological approaches for minimizing VILI

#### Neurally adjusted ventilatory assist

Neurally adjusted ventilatory assist (NAVA) delivers pressure to the airways proportional to inspiratory diaphragmatic electrical activity (EAdi); the proportionality factor is set on the ventilator by the clinician [20], via a specially designed nasogastric tube. EAdi is influenced predominantly by vagally-mediated feedback loops that integrate information from mechanoreceptors that ‘sense’ the degree of lung stretch, as well as chemoreceptors that sense chemical stimuli [21]. EAdi is reflexively upregulated if the delivered VT is below the patient’s respiratory demand, and downregulated if the assist is greater than the patient’s demand. When the assist level satisfies the patient’s respiratory demand, VT remains relatively constant despite increases in the proportionality factor. NAVA provides assist on a breath-by-breath basis in synchrony with, and in proportion to, the patient’s respiratory demand. This may be particularly effective in patients with increased work of breathing and/or respiratory muscle weakness. To the extent that a patient’s defense mechanisms are effective in limiting inappropriate lung stretch, NAVA may improve patient outcomes by tailoring MV to individual patient physiology, and its evolution over time (for example, during ARDS) [22].

Preclinical and clinical studies have demonstrated that NAVA can prevent excessive lung distention, efficiently unload respiratory muscles, and can improve patient-ventilator synchrony [20,23]. While on NAVA, innate reflexes in the lung will limit VT when the lungs get overstretched, allowing a patient to ‘control’ their own VT and limit VILI. This hypothesis was tested in an animal study in which 27 rabbits with hydrochloric acid-induced ARDS were randomized to NAVA or volume controlled MV with either low (6 ml/kg) or high (15 ml/kg) VT [24]. During NAVA, delivered VT was lower (mean 3.1 ml/kg), and PaO_2_/FiO_2_ ratio, respiratory rate, and PaCO_2_ were all significantly higher than in the 6 ml/kg control group. NAVA was similar to the low VT in preventing VILI, attenuating excessive systemic and remote organ inflammation, and in preserving cardiac and kidney function. Both NAVA and the 6 ml/kg volume group demonstrated reduced VILI and non-pulmonary organ dysfunction compared to the 15 ml/kg volume control group. This is the first study demonstrating that NAVA in spontaneously breathing animals with lung injury is similar to a conventional low VT strategy with regards to lung protection and non-pulmonary organ dysfunction. These promising results require confirmation in large, randomized controlled trial, since it is unclear whether the feedback mechanisms operative in the animal model can be translated to the much more complex human condition.

#### Targeting transpulmonary pressure and individualized PEEP titration

Transpulmonary pressure is the difference between alveolar pressure and pleural pressure, and is considered by some as the main determinant of VILI [25]. Alveolar pressure may be approximated from airway pressure under static conditions (that is, breath hold at end inspiration and end expiration). Since measurement of pleural pressure is invasive, esophageal pressure (Pes) is commonly used instead [26]. Disproportionate mechanical stress (that is, high transpulmonary pressure at end inspiration) on the injured lung is a key trigger for mechanotransduction and VILI. Atelectrauma may be mitigated by using adequate levels of positive end-expiratory pressure (PEEP) to prevent derecruitment at end expiration. Higher levels of PEEP have been shown to be lung protective in a number of animal models of ARDS [3], but have led to inconsistent results in clinical trials [27,28]. One possible explanation for the lack of apparent benefits in these clinical trials is the failure to tailor ventilatory support according to the patient’s physiology (that is, transpulmonary pressure), as the majority of these studies titrated PEEP according to a fixed protocol based on airway pressure and gas exchange parameters. Failure to account for the individual patient’s pleural pressure, and the recruitability of individual patient’s lungs may result in the underapplication or overapplication of PEEP, and an increased risk for VILI.

One reason why higher levels of PEEP have shown discordant results in animal models of ARDS as compared to clinical trials may be an increased contribution of a stiff chest wall [26] and high intra-abdominal pressure [29] in patients compared to the usual animals models of lung injury. Loring and colleagues [30] examined chest wall constriction at different levels of PEEP in an experimental lung lavage ARDS model. In the first experimental group, the animals had their chest walls constricted with an elastic binder and had a fixed PEEP (Group LC); the other group had the same constriction, but PEEP was raised to maintain the preconstriction level of end-expiratory transpulmonary pressure (Group LCP). The experimental groups were ventilated with the same strategy for 1.5 h after saline lung lavage, and had transpulmonary pressure measured using an esophageal balloon. Following MV, the LC group exhibited significantly worse lung mechanics, hypoxemia, and degree of pulmonary edema than either the LCP or the control groups. Proinflammatory cytokines in the blood and lung lavage fluid were elevated in all groups, with significantly higher in group LC. Group LC also had significantly worse histological signs of VILI than the other groups. Thus, maintaining transpulmonary pressure with additional PEEP could ameliorate the deleterious effects of chest wall constriction.

A pilot randomized clinical trial in patients with ARDS compared a strategy where PEEP was set to maintain a positive transpulmonary pressure measured by an esophageal balloon (intervention group), compared to a scale that titrated PEEP according to oxygenation (control group). The intervention group had improved respiratory system mechanics, oxygenation, and a non-significant survival advantage [31]. This study also demonstrated that an individualized ventilation strategy titrated according to transpulmonary pressure was feasible and could determine an appropriate level of PEEP. An ongoing multicenter, randomized control trial (EPVent 2; ClinicalTrials.gov NCT01681225) of MV directed by esophageal pressure measurement and maintaining a minimal but positive transpulmonary pressure throughout the ventilatory cycle will help to provide additional data on the potential efficacy of this individualized strategy in patients with ARDS.

### Lung recruitment maneuvers, high frequency oscillatory ventilation, and airway pressure release ventilation

Recruitment maneuvers (RMs) have been recommended as potential adjuncts to lung protective ventilation strategies. Tidal recruitment and subsequent derecruitment can occur even at low VT, leading to an increase potential for VILI [32]. Even with strict adherence to pressure and volume limited ventilation, up to a third of patients are exposed to end-inspiratory alveolar overdistention [33]. This phenomenon predominantly occurs in patients with a larger proportion of non-aerated lung, presumably because the VT is distributed into a much smaller aerated compartment. Recruiting non-aerated lung may attenuate this overdistension injury by distributing the VT more homogeneously, into a larger volume of aerated lung. RMs may be used to open collapsed, non-aerated lung units through a transient intentional increase in the transpulmonary pressure (for example, continuous positive airway pressure (CPAP) 40 cm H_2_O for 40 s) above that achieved by tidal ventilation [34].

There is evidence from animal models that infrequent RMs do not promote alveolar epithelial damage to the same extent as injurious, high pressure mechanical ventilation [35]. However, the volume of hyperinflated lung units does increase when a RM is performed (from 1% to 28% of lung volume) [36,37]. Indeed, the application of high transpulmonary pressures progressively increased the volume of open lung units that were hyperinflated, demonstrating the potential for worsening iatrogenic lung injury in a dose-dependent fashion [38]. Finally, laboratory studies have suggested that partial recruitment may aggravate cytokine production in the lung. The atelectatic lung is relatively inert and has little cytokine production, which may be markedly increased by inadequate recruitment or repeated derecruitment [39,40]. Thus, VILI may be further mitigated by opening, and keeping open, those unstable lung units that are cyclically collapsing, thus preventing atelectrauma. RMs do open the lung if applied early in the course of ARDS. They are useful in improving the PaO_2_/FiO_2_ and are sustainable if an appropriate PEEP is used post RM [41]. Nonetheless there are no convincing clinical data that RMs are useful in improving outcomes in patients with ARDS [42].

In addition to RMs, several recent ventilatory innovations may provide their benefit largely through progressive lung recruitment. These include high frequency oscillatory ventilation (HFOV), airway pressure release ventilation (APRV) [43]. HFOV should theoretically be an ideal mode to ventilate patients with severe lung damage [44]. It achieves gas exchange by delivering very small VT (often less than the anatomic dead space) at frequencies ranging from 3 to 5 Hz around a relatively constant mean airway pressure. Both these modalities use high, mean airway pressures to recruit and maintain adequate end-expiratory pressure while attenuating VILI. Similar to higher levels of PEEP, HFOV has been shown to maintain the oxygenation benefit from lung recruitment achieved with prone positioning [45]. Like RMs, both HFOV and APRV have been shown to improve oxygenation but lack a significant mortality benefit in the small number of clinical studies performed to date [43]. These ‘open lung’ approaches and their effects on VILI were compared to low VT ventilation in a study by Albert and colleagues [46]. Following surfactant depletion and injurious MV, 22 pigs were divided into 4 groups: low VT ventilation with 6 ml/kg PBW (low tidal volume ventilation (LTVV); n = 6); 2) HFOV (n = 5); 3) APRV (n = 6); and 4) RM (incremental PEEP) followed by decremental PEEP titration (RM; n = 5). Lung and hemodynamic parameters were evaluated every 30 minutes for 6 h following lung injury. Bronchoalveolar lavage fluid and lung tissue were analyzed for cytokines and histology, respectively. Oxygenation improved in all three open lung groups as compared to the LTVV group, significantly in the APRV and RM groups. IL-8 and TNFα were significantly lower in the APRV group as compared with the LTVV group, with no significant differences in cytokine concentrations between LTVV and the other two groups. Finally, APRV was associated with reduced histological markers of lung injury as compared to the LTVV group, whereas the HFOV and RM groups demonstrated greater airspace hemorrhage and leukocyte infiltration, respectively. Thus, none of the open lung techniques consistently and significantly reduced VILI as compared to LTVV. These experimental findings correlate with clinical studies that have failed to demonstrate a significant survival advantage with open lung strategies as compared to standard pressure-limited and volume-limited ventilation in ARDS [47,48]. Further study is needed to determine whether there is an appropriate therapeutic niche for these open lung strategies to reduce VILI and improve clinical outcomes in patients with ARDS.

### Extracorporeal gas exchange

Extracorporeal life support (ECLS) techniques, such as extracorporeal membrane oxygenation (ECMO) or extracorporeal CO_2_ removal (ECCO_2_R), can provide adequate gas exchange in patients with ARDS [49]. Vast improvements in ECLS technology over the last decade have made these devices less invasive, more biocompatible, and easier and safer to use. Moreover, ECLS can facilitate the use of ‘ultra’-protective MV (for example, employing VT <6 ml/kg PBW and lower airway pressures) in patients supported with ECLS, minimizing the risk of VILI. More radically, patients supported with ECLS may not require intubation or invasive MV at all: no ventilation, no VILI. Important additional benefits of this strategy may include decreased need for heavy sedation, with a concomitant decrease in delirium, and an increased ability to participate in early rehabilitation. Thus, ECLS may facilitate the awake, calm, cooperative, and mobile patient with ARDS, helping to ameliorate intensive care unit (ICU)-acquired weakness that contributes to the substantial and persistent morbidity in ARDS survivors [50]. Finally, promising results from recent case series during the H1N1 pandemic [51,52] and the Conventional Ventilatory Support versus Extracorporeal Membrane Oxygenation for Severe Adult Respiratory Failure (CESAR) trial [53] have led to renewed interest in ECMO as a strategy for managing severe ARDS in adults, even though there were a number of major methodological concerns and limitations with these studies.

In a proof-of-concept study, Terragni and colleagues [54] evaluated whether VT <6 ml/kg PBW may enhance lung protection. In 32 patients with ARDS ventilated with a VT of 6 ml/kg PBW, those with plateau pressures between 28 and 30 cm H_2_O had their VT reduced to achieve plateau pressures between 25 and 28 cm H_2_O. Respiratory acidosis (pH ≤7.25) was managed with ECCO_2_R for at least 72 h. Patients who already had plateau pressures between 25 and 28 cm H_2_O continued to receive MV with VT of 6 ml/kg PBW. In the ECCO_2_R group (ten patients), PaCO_2_ (mean 50 mmHg) and pH (mean 7.32) were normalized, and VT was reduced from 6 to 4 ml/kg PBW and plateau pressure decreased from 29 to 25 cm H_2_O (*P* <0.001). Moreover, there was a significant reduction in the morphological markers of lung injury and pulmonary cytokines (*P* <0.01) in the ECCO_2_R group after 72 h of MV with VT lower than 6 ml/kg PBW. Of note, no patient-related complications occurred in patients receiving ECCO_2_R.

While promising, the putative benefits of ‘ultra’-protective MV with ECCO_2_R, or more complete gas exchange support with ECMO, in patients with ARDS requires confirmation in large, randomized controlled trials. Moreover, the highly specialized equipment and knowledge required to provide ECLS make these techniques available only in specialized medical centers [55]. More definitive answers may be forthcoming from the ongoing multicenter randomized EOLIA (ECMO to rescue Lung Injury in severe ARDS; ClinicalTrials.gov NCT01470703) trial, which will compare venovenous ECMO to a modern protocolized lung protective ventilation strategy [56].

### Therapies to counteract biotrauma

#### Anti-cytokine therapy

MV can increase the level of inflammatory mediators within the lungs, and treatment with antagonists of these mediators may reduce VILI [3]. A number of potential targets have been identified in preclinical studies. Increased levels of several inflammatory mediators (including TNFα, IL-6, and IL-10) were found in *ex vivo* and *in vivo* rat models subjected to injurious mechanical ventilation [17,57]. It has been reported that IL-1 blockade mitigates inflammatory manifestations of VILI in animals [58,59], but this has not be investigated in humans. Other targets include the blocking or inhibition of IL-18 [60]. Hoegl and colleagues have recently reported that the prophylactic inhalation of anti-inflammatory cytokines, such as IL-10 and IL-22, may reduce or protect against VILI and improve survival [61,62].

After demonstrating significantly increased levels of the proinflammatory cytokine TNFα in the alveoli following MV in a saline lung lavage model of ARDS [63], Imai and colleagues [64] examined whether pretreatment with intratracheal anti-TNFα antibody would reduced the magnitude of VILI. They instilled low-dose (0.2 mg/kg; n = 6) or high-dose (1 mg/kg; n = 6) polyclonal anti-TNFα antibody in two treatment groups, as compared to a serum IgG fraction in the antibody control group (n = 6) and saline in the saline control group (n = 7). Following 4 h of MV, levels of TNFα in the lung lavage fluid were significantly higher than at baseline. Moreover, pretreatment with anti-TNFα antibody improved gas exchange and respiratory system compliance, reduced leukocyte infiltration, and ameliorated pathological findings in a dose-dependent fashion. However, given the complexity of the inflammatory response to MV and in ARDS, lung injury was not completely mitigated by pretreatment with anti-TNFα antibody in this model. Finally, it is important to note that despite promising results using animal models of VILI, to date clinical trials of anti-cytokine therapy in critically ill patients have not led to any significant demonstrable benefit [65,66]. While it is unlikely that a single anti-cytokine intervention for VILI will have a significant impact on patient-important outcomes, these therapies may represent useful adjuncts in the armamentarium against VILI in patients with ARDS, after appropriate evaluation in large clinical trials.

#### Heat shock response

First described as a response to thermal stress, the heat shock (or stress) response is characterized by the rapid expression of a highly conserved group of proteins (heat shock proteins) that can be induced by many thermal and non-thermal stressors, as well as various pharmacological agents [67]. The heat shock response is a very primitive defense mechanism present in all eukaryotic cells, including inflammatory cells and their target cells. In general, heat shock proteins are molecular chaperones that help to maintain cellular homeostasis by facilitating the proper folding, assembly, and stabilization of new and damaged proteins. Whatever the inciting stimulus, the heat stress response confers protection for subsequent thermal and non-thermal cytotoxic stressors. Although our understanding of the regulatory events governing the stress response and the mechanisms of protection are still limited, a number of studies suggest that this natural cytoprotective response may be a novel therapeutic strategy for the treating a number of inflammatory disease states. *In vitro* experiments have demonstrated that induction of the heat shock response protects against endotoxin-mediated apoptosis, peroxynitrite, and hydrogen peroxide, while *in vivo*, it protects animals against sepsis, ARDS, and ischemia-reperfusion injury [67].

One possible mechanism by which the heat shock response may be protective against subsequent stressors may be by binding of heat shock proteins to cytokines, preventing their release from inflammatory cells [68]. To examine the implications of this hypothesis in the setting of VILI, Ribeiro and colleagues [68] randomized experimental rats to either receive exposure to heat (rectal temperature 41°C for 15 minutes) or sham treatment. The lungs were harvested and either lavaged for cytokine analysis (pre-ventilation data) or mechanically ventilated with high VT for 2 h, and then lavaged for analysis (post-ventilation data). MV in sham treatment lungs led to a 47% reduction in compliance and an increase in lung lavage levels of proinflammatory cytokines (TNFα, IL-1β, macrophage inflammatory protein 2) as compared to low levels of cytokines in the pre-ventilation state. In contrast, MV in the heat stress group led to a smaller reduction in compliance (17%), and a significant attenuation of proinflammatory cytokines as compared to the post-ventilation state in the sham treatment group. Increased activation of nuclear factor κB, an important transcription factor of many inflammatory cytokines, has been reported to correlate with mortality in patients with sepsis. Recent studies have demonstrated that induction of the heat shock response inhibits nuclear translocation of nuclear factor κB in cultured respiratory epithelial cells [69]. Coupled with data demonstrating a reduction in organ dysfunction and mortality in sepsis-induced lung injury [70], induction of heat shock proteins and the heat shock response may prove to be a useful therapy to help attenuate or prevent VILI and its downstream consequences in patients with ARDS.

#### Future directions and conclusions

Despite four decades of research into ARDS, no specific therapies exist and the mortality remains unacceptably high. MV remains the cornerstone of supportive care, and the understanding that MV itself could lead to further lung injury (VILI) has been an important advance. Ventilatory strategies aimed at reducing the potential for VILI, by reducing the mechanical stress placed on the injured lung, have led to important reductions in mortality in patients with ARDS. Novel physiological approaches for mitigating VILI by limiting the mechanical stress applied to the injured lungs, such as NAVA and targeting transpulmonary pressure with individualized PEEP titration, represent the evolution of current lung protective strategies tailored to individual patients’ respiratory system physiology. Perhaps the ultimate therapy in the quest to limit airway pressures and VT to reduce VILI in patients with ARDS is the use of extracorporeal gas exchange to facilitate ‘ultra’-protective MV, or to obviate the need for intubation and MV all together. Without mechanical ventilation, there is no VILI, although lung injury can occur from spontaneous ventilation with very large tidal volumes [71]. In addition, the provision of ECCO_2_R and/or ECMO is associated with a number of risks and potential complications. Finally, these interventions may have synergistic effects when used in combination (for example, NAVA and ECCO_2_R), but their efficacy, alone or in combination, require confirmation in future clinical trials.

A potentially important paradigm shift for the treatment of VILI may include novel therapies aimed not only at decreasing pressures or volumes in the lung, but also directed at the development of interventions that are aimed directly at preventing the initiation and/or propagation of the inflammatory response (biotrauma). Anti-cytokine therapies and manipulation of the heat shock response may represent promising adjunctive therapies to both existing and novel lung protective ventilatory strategies to help obviate the development of VILI. Unfortunately, many promising therapies found to reduce VILI and improved outcomes in preclinical studies have not been translated into success in subsequent clinical trials in humans. As a result, ongoing preclinical and clinical investigations continue in hopes of addressing the reasons for these discrepancies and providing a better understanding of the complex interface between MV, the injured lung, and the critically ill patient. These studies will aid in developing novel approaches to ameliorate or mitigate the development of VILI.

## Abbreviations

ARDS: Acute respiratory distress syndrome; EAdi: Inspiratory diaphragmatic electrical activity; ECCO2R: Extracorporeal carbon dioxide removal; ECMO: Extracorporeal membrane oxygenation; ECLS: Extracorporeal life support; LTVV: Low tidal volume ventilation; MV: Mechanical ventilation; NAVA: Neurally adjusted ventilatory assist; PBW: Predicted body weight; PEEP: Positive end-expiratory pressure; RM: Recruitment maneuver; VILI: Ventilator-induced lung injury; VT: Tidal volume.

## Competing interests

EF has not disclosed any potential conflicts of interest. JV has received research grants from Maquet. AS received consultancy fees, educational grants, or served on advisory boards for Apeiron, Asthmatx, Broncus, GlaxoSmithKline, Leo Pharma, Lilly, Maquet, Novalung, Sensormedics, and Tarix.

## Authors’ contributions

All authors contributed equally to the inception and execution of this article and read and approved the final manuscript.

## Pre-publication history

The pre-publication history for this paper can be accessed here:

http://www.biomedcentral.com/1741-7015/11/85/prepub
